# Primary pulmonary colloid adenocarcinoma: A case report of a rare subtype

**DOI:** 10.1016/j.ijscr.2024.110260

**Published:** 2024-09-14

**Authors:** Ryusei Yamada, Nobuyuki Oguri, Fumiya Kawano, Mayu Inomata, Yuichiro Sato, Ryo Maeda

**Affiliations:** a Department of Thoracic and Breast Surgery, Faculty of Medicine, University of Miyazaki, Miyazaki, Japan; b Department of Diagnostic Pathology, Faculty of Medicine, University of Miyazaki Hospital, Miyazaki, Japan

**Keywords:** Case report, Colloid adenocarcinoma, Lung cancer, Recurrence, Metastasis

## Abstract

**Introduction and importance:**

Pulmonary colloid adenocarcinoma is an extremely rare subtype of lung adenocarcinoma. Owing to its rarity, the detailed clinical features of colloid adenocarcinoma remain largely unknown. This report describes a case of early-stage colloid adenocarcinoma that recurred soon after resection, including its radiological findings.

**Case presentation:**

During a routine checkup, a chest roentgenogram revealed an abnormal shadow in the right upper lung field of an asymptomatic 68-year-old man. Computed tomography (CT) showed a well-defined, low-attenuation nodule in the right upper lobe. Right upper lobectomy with mediastinal lymph node dissection was performed. The postoperative histopathological diagnosis indicated pulmonary colloid adenocarcinoma. The pathological stage was classified as T1bN0M0 (stage IA2). Follow-up CT 1 year after the resection revealed an enlarged supraclavicular lymph node and pulmonary nodule in the right lower lobe. Both lesions appeared as well-defined solitary hypoattenuated tumors with minimal enhancement on CT images. Excisional biopsies of both tumors were performed to obtain a definitive diagnosis. Both tumors consisted of abundant mucin in which some tumor cells were floating and were diagnosed as colloid adenocarcinoma recurrences.

**Clinical discussion:**

Although colloid adenocarcinoma is generally considered to have indolent clinical behavior, it can recur even in early-stage cases.

**Conclusion:**

Colloid adenocarcinoma is a distinct variant of lung adenocarcinoma, characterized by well-circumscribed mucinous lesions with alveolar wall destruction caused by mucin pools and scant tumor cells. The treatment strategy for colloid adenocarcinoma should follow the guidelines for primary lung cancer.

## Highlights

• Pulmonary colloid adenocarcinoma is a rare subtype of lung adenocarcinoma.
• Although colloid adenocarcinoma is considered to have an indolent clinical behavior, it can recur even in early-stage cases.

## 1 Introduction

Pulmonary colloid adenocarcinoma is an extremely rare subtype of lung adenocarcinoma newly classified as a variant by the 2015 World Health Organization (WHO) classification [1]. Although colloid adenocarcinoma is considered to have an indolent clinical behavior compared to conventional lung adenocarcinoma [2,3], detailed clinical features of pulmonary colloid adenocarcinoma remain unknown due to its rarity. In this report, we describe an early-stage pulmonary colloid adenocarcinoma that recurred soon after complete resection, along with its radiological findings. Additionally, we present a review of pulmonary colloid adenocarcinoma cases (*n* = 39), including the present case, reported in the English literature to elucidate its clinical behavior [[4], [5], [6], [7], [8], [9]]. This study was conducted in accordance with the principles of the Declaration of Helsinki and the SCARE 2023 guidelines [10].

## 2 Case presentation

**Fig. 1:**
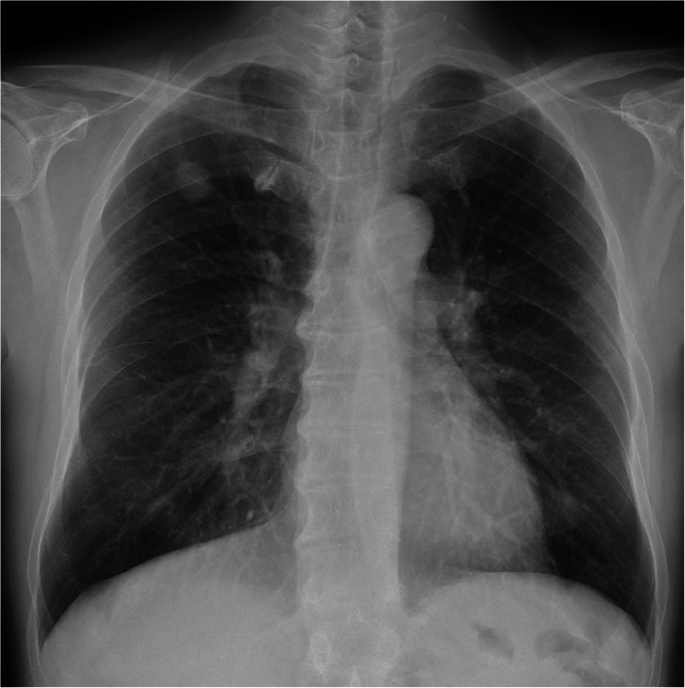
Chest roentgenogram revealing a well-defined nodule in the right upper lung field.

**Fig. 2:**
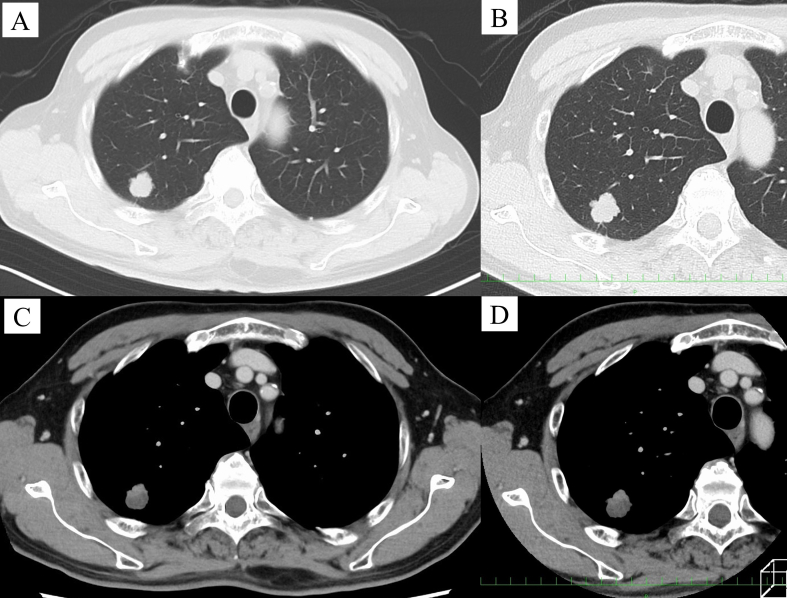
(A-D) Computed tomography images showing a well-defined, low-attenuation lobulated nodule in the right upper lobe.

During a regular checkup, chest roentgenography revealed an abnormal shadow in the right upper lung field of an asymptomatic 68-year-old man (Fig. 1). Computed tomography (CT) showed a well-defined, low-attenuation lobulated nodule measuring 2.3 × 2.1 cm in the right upper lobe (Fig. 2A-D). Hematological examination results, including tumor markers such as a carcinoembryonic antigen, cytokeratin fragment 21, and pro-gastrin-releasing peptide, were within normal ranges. Although a transbronchial lung biopsy was unsuccessful, the nodule was highly suspicious of lung cancer. No apparent tumor was detected by abdominal CT, brain magnetic resonance imaging, or bone scintigraphy. Thus, his clinical stage was classified as T1cN0M0, stage IA3 disease. We performed right upper lobectomy and lymph node dissection in the hilum and mediastinum using video-assisted thoracic surgery (VATS).

**Fig. 3:**
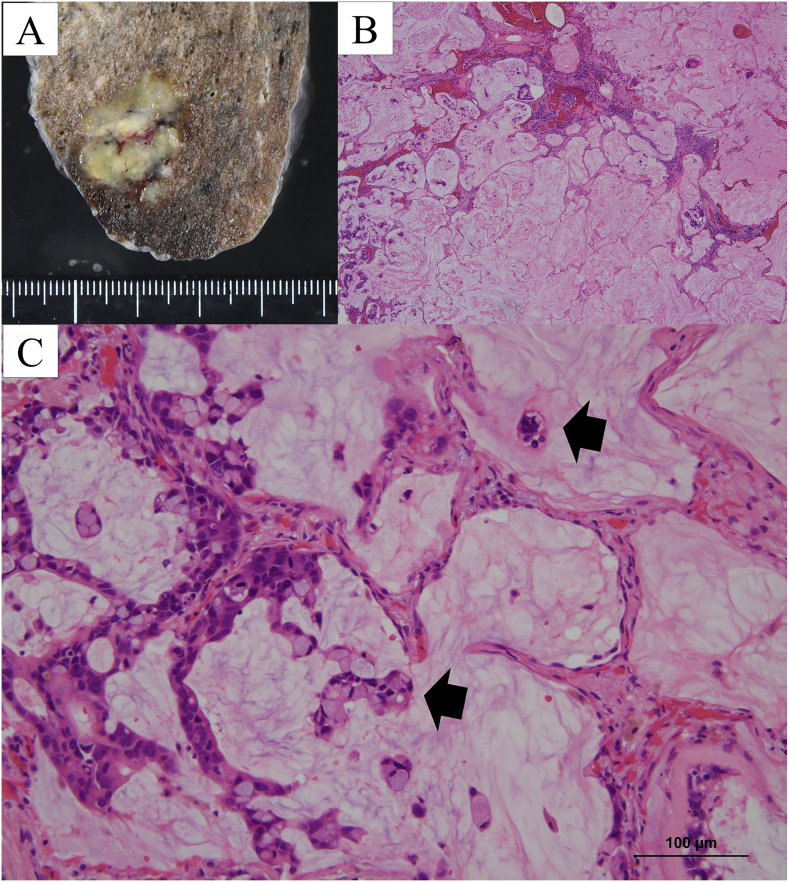
(A) The cut surface of the resected specimen showing a lesion with mucin pooling. (B) Tumor tissues demonstrating rupture of the alveolar septum and abundant mucin, which forms mucous pools (Hematoxylin and Eosin [HE] stain, ×40). (C) Some tumor cells clustering and floating in the mucous pools (black arrow) (HE stain, ×200).

The cut surface showed a well-demarcated nodule measuring 2.0 cm in maximum diameter, filled with a yellowish-white gelatinous substance (Fig. 3A). Histopathologically, the tumor consisted of abundant mucin filling the alveolar spaces with some tumor cells floating in the mucin pools (Fig. 3B-C). The alveolar walls were destroyed by abundant extracellular mucin. Postoperative histopathological findings were consistent with the diagnosis of pulmonary colloid adenocarcinoma. The pathological stage was classified as T1bN0M0 (stage IA2). The patient was discharged uneventfully on the fourth postoperative day.

**Fig. 4:**
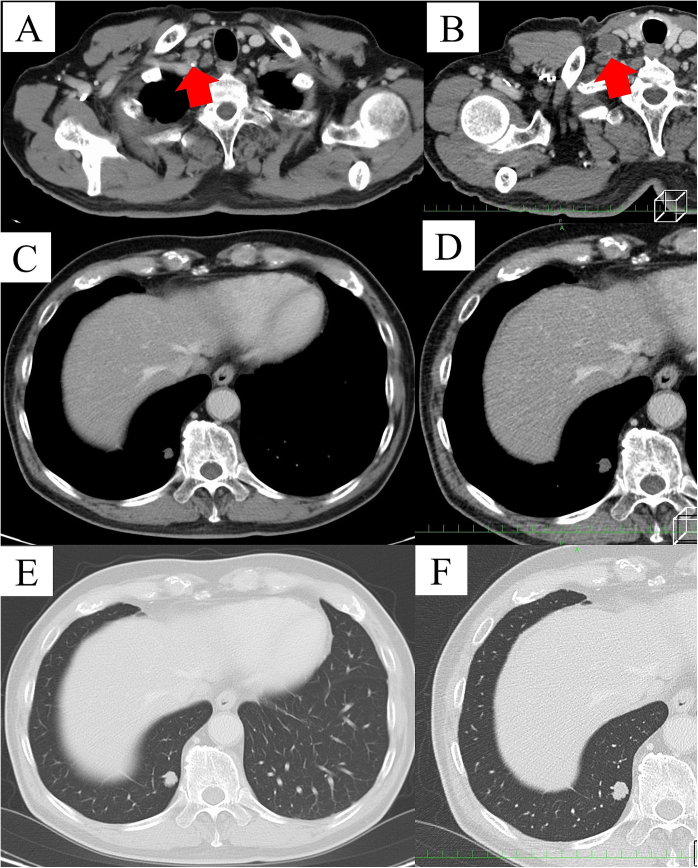
(A-B) Thoracic computed tomography (CT) images revealing an enlarged supraclavicular lymph node with low attenuation and poor enhancement (red arrow). (C-F) CT images showing a well-defined solitary hypoattenuated tumor with minimal enhancement in the right lower lobe. (For interpretation of the references to colour in this figure legend, the reader is referred to the web version of this article.)

**Fig. 5:**
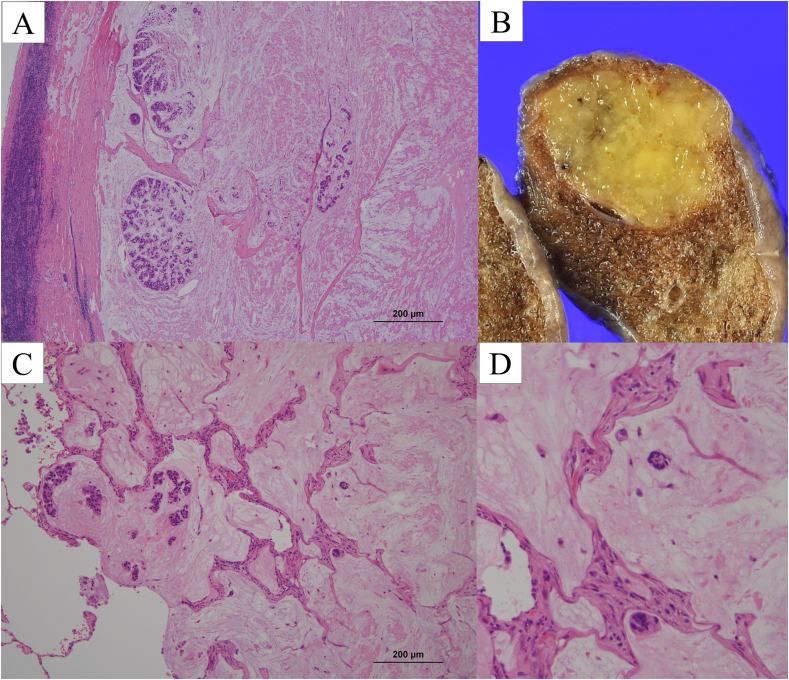
(A) Microscopic findings of the supraclavicular lymph node in the specimen, showing lesion with a rich mucinous pool and a small number of tumor cells floating within it (Hematoxylin and eosin (HE) stain, ×40). (B) Cut surface of the resected specimen of the nodule from the right lower lobe, showing a lesion with mucin pooling. (C-D) Microscopic findings of the right lower lobe nodule showing a tumor with a rich mucinous pool, and both single and clustered cancer cells float within it (HE stain).

Although no recurrence was observed by chest CT six months after the resection, follow-up CT 1 year after the resection revealed enlarged lymphadenopathy at the base of the right supraclavicular region (Fig. 4A-B) and a pulmonary nodule measuring 9 mm in the right lower lobe (Fig. 4C-F). Both lesions appeared as well-defined solitary hypoattenuated tumors with little enhancement on CT images. Excisional biopsy of the supraclavicular lymph node and partial resection of the right lower lobe using VATS were performed to obtain a definitive diagnosis. Both tumors consisted of abundant mucin with some floating tumor cells and were diagnosed as recurrences of colloid adenocarcinoma (Fig. 5A-D). Mutations in the epidermal growth factor receptor (EGFR), Kirsten rat sarcoma viral oncogene homolog (KRAS), and anaplastic lymphoma kinase (ALK) genes were not found. Additionally, assessment of programmed death-ligand 1 (PD-L1) expression using the antibody 22C3 (Dako PharmDx) demonstrated a tumor proportion score of less than 1 %. Although the patient received four cycles of carboplatin, pemetrexed, and pembrolizumab, numerous metastatic lesions, including multiple lung metastases and multiple cervical lymph node metastases, had emerged.

## 3 Discussion

The variants of lung adenocarcinoma in the 2015 WHO classification include invasive mucinous adenocarcinoma, colloid adenocarcinoma, fetal adenocarcinoma, and enteric adenocarcinoma [1]. In this classification, colloid adenocarcinoma was newly recognized as a variant of lung adenocarcinoma [1]. In the past, colloid adenocarcinoma had various names [2,3,[11], [12], [13], [14]]. The term “mucinous (colloid) adenocarcinoma” in the 2007 WHO classification referred to an “enteric adenocarcinoma” with mucin pools, which differs from the current definition of “colloid adenocarcinoma” [1,8]. According to the latest WHO classification, colloid adenocarcinoma is histologically defined as an adenocarcinoma with abundant extracellular mucin in pools, which distends alveolar spaces and destroys their walls [1].

Histologically, distinguishing colloid adenocarcinoma from invasive mucinous adenocarcinoma of the lung can be challenging. In invasive mucinous adenocarcinoma [1], the tumor cells are predominantly goblet-shaped or columnar, adhere to the wall, and exhibit multifocal and leaping growth. However, the alveolar structure remains intact. In contrast, the present case was diagnosed as a colloid adenocarcinoma based on the destruction of the alveolar walls due to large amounts of mucus secreted by the tumor cells, which were floating in a mucous lake.

Imaging findings of colloid adenocarcinomas have occasionally been described in the radiology literature [6]. Most of these reports, including the present case, have shown a well-defined solitary tumor with low attenuation on CT due to a large amount of mucus [6,7,9]. In contrast, invasive mucinous adenocarcinoma typically presents as pneumonia or consolidation on radiological imaging and displays mucoid contents on gross (anatomical) examination [15,16]. In our study, the recurrence lesions, including a supraclavicular lymph node and lung metastasis, also appeared as well-defined solitary hypo-attenuated tumors with minimal enhancement on CT images. Both recurrent sites contained a large amount of mucus histopathologically. Invasive preoperative diagnostic procedures, such as transbronchial biopsy and transthoracic needle biopsy, are generally inadequate for histological diagnosis due to tumor composition (i.e., large amounts of mucin and a small number of malignant cells) [7]. Consequently, making a diagnosis preoperatively and even intraoperatively can be challenging [7]. Given that the recurrence sites also contained substantial amounts of mucus in the present case, a needle biopsy is insufficient; an excisional biopsy is needed for an accurate diagnosis of recurrence.

Pulmonary colloid adenocarcinoma is extremely rare, accounting for 0.13 % of all primary lung cancers [5]. Owing to its rarity, the detailed clinical features of pulmonary colloid adenocarcinoma remain largely unknown. Since its establishment in the 2015 WHO classification [1], 38 cases of colloid adenocarcinoma have been reported in the English literature [[4], [5], [6], [7], [8], [9]]. We have summarized 39 cases of colloid adenocarcinoma, including the present case, accompanied by a literature review (Table 1, Table 2). The age of patients with pulmonary colloid adenocarcinoma ranged from 33 to 86 years, with a mean age of 66 years. Of the 39 patients, 20 (51 %) were male. The mean tumor size was 3.5 cm (range, 1.5–8.2 cm). Owing to its well-circumscribed margin and bland-looking cytology, some authors have refrained from using the term “carcinoma.” Many early publications classified these tumors as benign, low-grade, or borderline neoplasms. However, of the 39 patients, 9 (23 %) experienced tumor recurrence within the follow-up period (range, 1.9–128 months) (Table 2). In addition, 9 (23 %) out of the 39 patients had lymph node metastases (Table 2). Colloid adenocarcinoma is a malignant tumor and can be characterized by lymph node metastasis. The treatment strategy for colloid adenocarcinoma should be based on the guidelines for primary lung cancer. Lobectomy or segmentectomy with lymph node dissection is considered the standard surgical approach for colloid adenocarcinoma. Of the 39 cases, 18 (46 %) were diagnosed as stage IA. Although our case recurred soon after surgery, none of the other cases had tumor recurrence within the follow-up period (range, 1.9–120 months). However, care should be taken to follow the postoperative course of patients as conventional lung adenocarcinoma, even in cases diagnosed as stage IA.

**Table 1:** Characteristics of the colloid adenocarcinoma cases reported in the English literature.

	No. of Cases (%)
Overall number	39
Age (years)	
Mean (Range)	65 (33–86)
Sex	
Male	20 (51)
Female	19 (49)
Tumor sizes (mm)	
Mean (Range)	35 (15–82)
Localization	
Unknown	13
Right upper lobe	7
Right middle lobe	5
Right lower lobe	5
Left upper lobe	3
Left lower lobe	6
Extent of resection	
Unknown	17
Wedge resection	6
Lobectomy	16
Pathological stage	
IA	
IA1	4 (10)
IA2	6 (15)
IA3	8 (21)
IB	6 (15)
IIA	3 (8)
IIB	6 (15)
III	6 (15)

**Table 2:** Clinical outcomes of the colloid adenocarcinoma cases reported in the English literature.

	No. of cases	Pathological stage		
		IA	IB	II or III
Total	39	18	6	15
Lymph node metastasis				
N0	30	18	6	6
N1	3	0	0	3
N2	6	0	0	6
Follow up (months)				
Mean (Range)	38 (1.9–128)	27 (1.9–120)	21 (13–61)	40 (10.5–128)
No. of recurrence	9	1	1	7

Regarding the EGFR/ KRAS/ ALK mutation status, no mutations in these genes were found in the present study. According to Zenali et al. [4], 2 out of 13 patients had a KRAS mutation in their tumors. This frequency is relatively similar to that of conventional pulmonary adenocarcinoma but appears lower than those reported in previous studies of mucinous-type adenocarcinoma [17,18]. Further studies are required to identify specific gene alterations other than KRAS mutations in colloid adenocarcinoma.

## 4 Conclusion

Colloid adenocarcinoma is a distinct variant of lung adenocarcinoma, characterized by well-circumscribed mucinous lesions with alveolar wall destruction due to mucin pools and scant tumor cells. Although colloid adenocarcinoma is generally considered to have an indolent clinical behavior, tumor recurrence can occur even in early-stage cases. The treatment strategy for colloid adenocarcinoma should follow the guidelines for primary lung cancer.

## Abbreviations

WHO: World Health Organization
CT: Computed tomography
VATS: video-assisted thoracic surgery
EGFR: epidermal growth factor receptor
KRAS: Kirsten rat sarcoma viral oncogene homolog
ALK: anaplastic lymphoma kinase
PD-L1: programmed death-ligand 1

## CRediT authorship contribution statement

Dr. Ryusei Yamada has designed this report.

Dr. Nobuyuki Oguri and Dr. Yuichiro Sato have provided us with the histological diagnosis and photos of the slides, and have reviewed.

Dr. Fumiya Kawano and Dr. Mayu Inomata have reviewed.

Dr. Ryo Maeda is the writer of this article and corresponding author.

## Consent

Written informed consent was obtained from the patient for publication of this case report and accompanying images. A copy of the written consent is available for review by the Editor-in-Chief of this journal on request.

## Ethics approval

As it is a case report, ethical approval is exempted by University of Miyazaki Hospital.

## Funding

The authors have no competing interests to declare.

## Research registration number

Not applicable.

## Guarantor

Dr. Ryo Maeda accepts all responsibility of this article.

## Declaration of competing interest

All author declare that they have no conflicts of interest.
